# News about Structure and Function of Synapses: Health and Diseases

**DOI:** 10.3390/biomedicines10102596

**Published:** 2022-10-17

**Authors:** Jacopo Meldolesi

**Affiliations:** 1 San Raffaele Institute, Vita-Salute San Raffaele University, 20132 Milan, Italy; meldolesi.jacopo@hsr.it; 2 CNR Institute of Neuroscience, Milan-Bicocca University, 20132 Milan, Italy

During the last century, synapses have been intensely investigated as the most interesting sites of neuroscience development. However, knowledge about many of these studies has not reached rapid completion, but rather still requires a continuous updating. Moreover, the important aspects, critical for basic and/or medical functions, have remained largely separate. The present Special Issue about Synapses intends to combine dual distinct fields: the basic functions at present are intensely discussed and innovative synaptic documents about neurodegenerative diseases and their properties are presented. The present SI offers common properties by updating the aspects of synapses which are relevant for both physiology and disease states. In other words, the SI illustrates how exciting synapses are today for their recently discovered basic and medical properties, and for the expectation that they will have at least some developments in the next few years.

Our SI presentation includes five reviews and one brief report of general significance. All authors are well-known specialists in the various areas.

The expert on the synaptic functions, Sumuko Mochida from Tokyo Medical University, illustrated the growing functions of synaptic active zone proteins, from their participation in various aspects of presynaptic plasticity to activity-dependent endocytosis [1].

Leonid P. Savichenko and Dmitri A. Rusakov, from University College London, London, developed new, model-revealed data on glutamate distribution at and near the synapses. Such a distribution depends on several processes related to the synaptic conditions: release, diffusion and uptake by the high affinity transporters of the surrounding astroglia [2]. 

My own review about brain postsynaptic structures emphasized both the compositional and functional differences between flat and spines. This difference accounts for their association with inhibitory and stimulatory synapses, respectively. Moreover, the activation of spines can induce a back regulation of the corresponding presynaptic functions [3].

The innovative properties of synapses in neurodegenerative diseases are provided by three contributions.

Fabrizio Gardoni, Monica Diluca, working with Maria Italia and Elena Ferrari from the Milan State University, worked on two critical misfolded proteins, tau and α-synuclein, affecting the function of NMDA and AMPA glutamatergic receptors. The results revealed the role of this approach in various specific diseases [4].

Berenice A. Gutierrez and Agenor Limon, from the University of Texas in Galveston, started from the severity induced by soluble oligomer seeding of neuropathological hallmarks. In Alzheimer’s and Parkinson’s diseases, oligomers of amyloid β and of α-synuclein were found to induce severe lesions up to the synaptic disruption and the final loss. In the oligomer’s absence, cognition was preserved [5].

Arianna Bellucci, Francesca Longhena and Maria Grazia Spillantini from Cambridge University realized that synaptic damage finally induces neurodegeneration up to neuronal loss. Interestingly, the synaptic architecture and function depend on the Rab small G protein family. Upon vesicle aggregation by α-synuclein, Rabs participate in synaptic damage [6].

Our six, largely innovative contributions are most likely of interest for specialists in the various fields. Their participation in a qualified SI is expected to stimulate people’s attention for the problems discussed in the adjacent presentations, increasing their curiosity and resulting in innovative developments.
